# A Cross-Sectional Study of Serum Glutathione Peroxidase: An Antioxidative Marker in Chronic Periodontitis and Chronic Kidney Disease

**DOI:** 10.7759/cureus.22016

**Published:** 2022-02-08

**Authors:** Plato Palathingal, Jaideep Mahendra, Pandapulaykal T Annamalai, Shyam S Varma, Little Mahendra, Libby Thomas, Deepak Baby, Akhil Jose, Sruthi Srinivasan, Ambily R

**Affiliations:** 1 Periodontics, PSM College of Dental Science and Research, Thrissur, IND; 2 Periodontics, Meenakshi Ammal Dental College and Hospital, Chennai, IND; 3 Biochemistry, Jubilee Mission Medical College and Research Institute, Thrissur, IND; 4 Nephrology, Jubilee Mission Medical College and Research Institute, Thrissur, IND; 5 Periodontics, Maktoum Bin Hamdan Dental University College, Dubai, ARE; 6 Periodontics, Al Zahir Medical Complex, Salalah, OMN; 7 Conservative Dentistry and Endodontics, PSM College of Dental Science and Research, Thrissur, IND; 8 Pedodontics, PSM College of Dental Science and Research, Thrissur, IND; 9 Periodontology, Meenakshi Ammal Dental College and Hospital, Chennai, IND; 10 Microbiology, College of Veterinary and Animal Sciences, Thrissur, IND

**Keywords:** renal disease, chronic periodontitis, biomarker, systemic interactions, comorbidities, infection, oxidative stress, serum glutathione peroxidase

## Abstract

Background and aim: Oxidative stress as an individual risk for periodontitis and chronic kidney disease (CKD) has been elaborated through various mechanical pathways, yet its role in association with both diseases remains unexplored. Thus, the current study aims in evaluating serum glutathione peroxidase, an oxidative stress marker in CKD patients with periodontitis, and compare it with the healthy controls.

Methodology: One hundred and twenty subjects were divided into four groups as control (C=30 subjects), periodontitis and non-CKD patients (CP=30 patients), non-periodontitis and CKD patients (CKD=30 patients), and periodontitis and CKD patients (CKD+CP=30 patients). Demographic variables, periodontal parameters, such as plaque index (PI), gingival index (GI), probing pocket depth (PPD), percentage proportion of sites with probing pocket depth more than 5 mm, clinical attachment loss (CAL), percentage proportion of sites with clinical attachment loss more than 3 mm and serum stress marker, and glutathione peroxidase were compared between the groups and the results were statistically analyzed.

Results: The demographic variables did not differ significantly between the groups, except for age. The means PI, GI, PPD, percentage proportion of sites with probing pocket depth more than 5 mm, CAL, percentage proportion of sites with clinical attachment loss were higher in CKD+CP. The glutathione peroxidase was significantly higher in CP group (p=0.001) and significantly correlated with periodontal parameters.

Conclusion: The oxidative stress marker glutathione peroxidase was higher in CP, followed by the CKD groups. This could pave a strong link of oxidative stress as a risk factor for chronic periodontitis, as well as chronic kidney disease.

## Introduction

Chronic kidney disease (CKD) is a worldwide disease, with Asians displaying a high prevalence compared to other countries that adversely affects the patient’s quality of life which later gets associated with high morbidity and mortality. The antioxidant mechanisms are the evolutionary designs that avidly react with and initiate reactive oxygen species (ROS) before they inflict oxidative damage to the tissues and cells [1,2]. Chronic periodontitis is a disease caused by Gram-negative bacteria that destroy the supporting tissues of the teeth, induce local inflammation, and are associated with a systemic inflammatory response and oxidative stress [2].

Glutathione peroxidase (GPx) is one of the enzymes that play an important role in host defense against oxidative stress in cytosol [3]. Glutathione peroxidase is a selenium-containing enzyme that detoxifies hydrogen peroxide and various hydroperoxides using glutathione as a reducing agent [4]. Plasma glutathione peroxidase, an important extracellular antioxidant, is produced mainly in the kidney and has been detected in numerous human fluids [4].

When there is an imbalance, caused by a reduction in antioxidant defense and/or ROS production or activity, oxidative stress results [5]. Prognostic factors associated with worse outcomes in CKD include severity of kidney disease, systemic inflammation, and oxidative stress. The removal of ROS by antioxidant defense systems is essential for maintaining health [6-8]. The oxidative stress markers have been viewed in various inflammatory diseases, however, their role in periodontitis patients with CKD has not been elucidated so far. Glutathione peroxidase has shown to be a promising antioxidative stress marker instigating the pathological phenomena in relation to both the diseases.

Thus, the current study aimed to assess the demographic variables, periodontal parameters, and evaluate the glutathione peroxidase levels in periodontitis patients with and without CKD and compare them with the non-periodontitis patients with and without CKD, thus confirming their significance of association/correlation in periodontitis, CKD, and a combination of both in a representative sample of the Indian population.

## Materials and methods

This study was performed as a cross-sectional study that conformed with Strengthening the Reporting of Observational Studies in Epidemiology (STROBE) guidelines for reporting observational studies. The study was conducted from January 2019 to February 2020 in Thrissur, Kerala, India. A total of 180 patients (both CP and CKD) were recruited from PSM College of Dental Science and Research, Thrissur, India. CKD patients visiting PSM General Hospital, Thrissur, for their master heath checkup were selected for the study.

**Figure 1:**
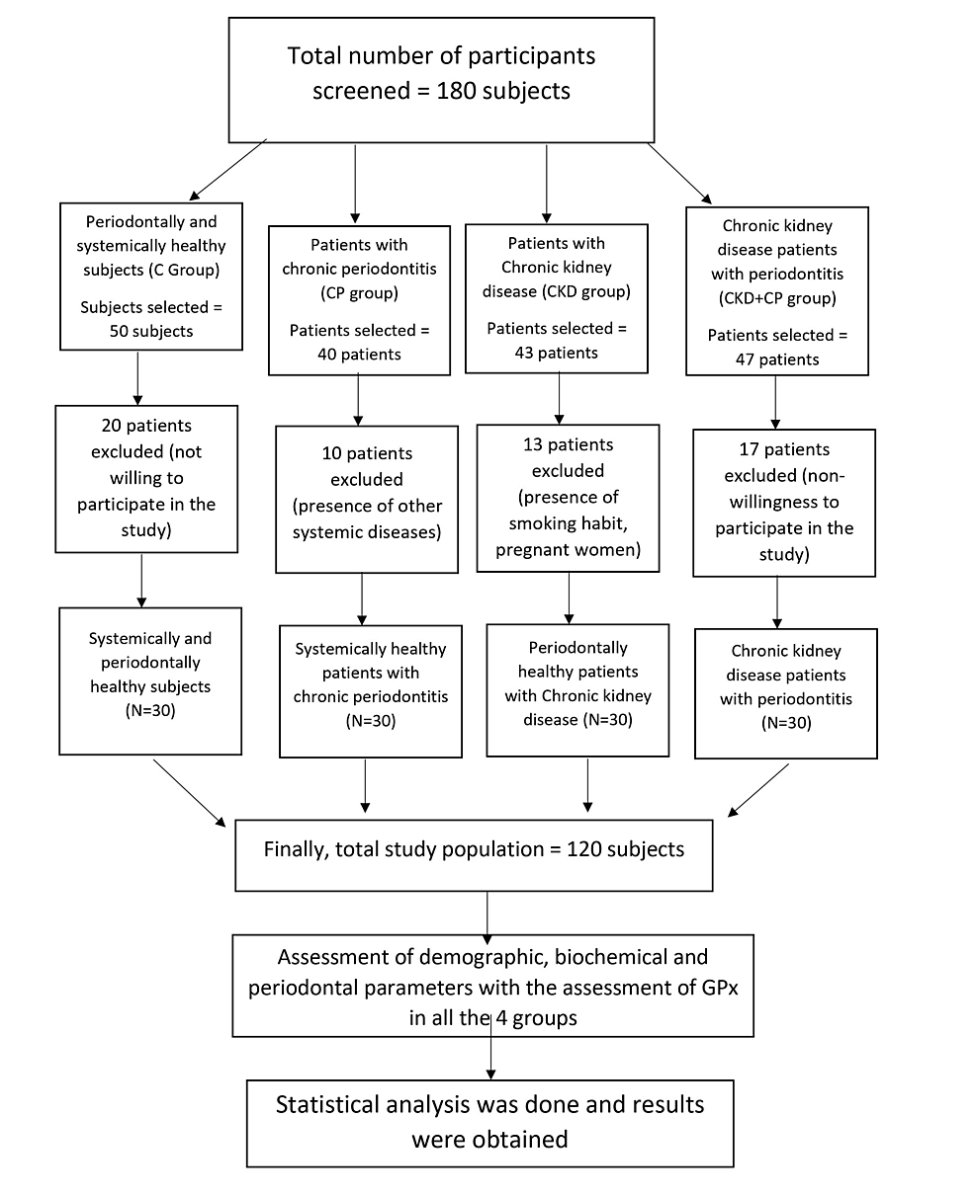
Study design flowchart.

In our study, subjects were categorized into four groups. Forty patients were diagnosed with chronic periodontitis and did not have any kidney problems. Among them, 10 patients were excluded due to the presence of other systemic diseases (e.g., cardiovascular disease, diabetes, etc.). Hence, 30 patients were recruited finally into chronic periodontitis (CP) group. Forty-three periodontally healthy patients with chronic kidney disease (stage 2-4) were categorized into the CKD group. Among them, 13 were excluded owing to their smoking habits and pregnancy. Thus, finally, only 30 patients were included in CKD group. Forty-seven patients with chronic kidney disease (stage 2-4, with eGFR<90 mL/min) and chronic periodontitis were recruited into the CKD+CP group. However, among them, 17 were excluded as they were not willing to participate in the study, which derived to a final number of 30 patients in CKD+CP group. Fifty subjects who were periodontally and systemically healthy were recruited, out of which 30 subjects were selected for the control group (C) group. Hence, the final study population included was 120 subjects (Figure 1). The patient selection was based on the following inclusion and exclusion criteria.

Inclusion criteria for the selected patients included (a) patients willing to participate in the study; (b) patients within 35-75 years of age group; (c) male and female patients; (d) patients with minimum of 12 natural teeth; (e) in case of CKD and C groups, individuals who were with healthy periodontium; (f) in case of CP and CKD+CP groups, individuals with periodontitis satisfying the case definition by 2017 classification of periodontal and peri-implant diseases and CKD stage 2-4 and not on dialysis were selected; and (g) in CP and C groups, patients presented with glomerular filtration rate >90 mL/min, estimated according to Chronic Kidney Disease Epidemiology Collaboration (CKD-EPI) 2009 [9].

The exclusion criteria included (a) patients who had undergone periodontal therapy within the previous six months; (b) patients with smoking/tobacco/alcohol habits; (c) sarcoidosis patients; (d) carcinoma patients; (e) rheumatoid arthritis patients; (f) immunosuppressive conditions; (g) tuberculosis patients; (f) patients with other inflammatory diseases; and (g) the subjects with eGFR<15 mL/min/1.73 m^2^. The patients were excluded due to high chances of being on dialysis or being seriously ill with a low response rate.

The power and confidence interval of the study was calculated to be 80% and 95%, respectively with 30 samples in the four groups based on the hospital records. This study was approved by the Institutional Ethical Review Board of PSM College of Dental Science and Research, Thrissur, India (protocol no: PSMDC/IEC/01/2015). The population of this cross-sectional study was selected from patients recruited from the Department of Periodontics in PSM College of Dental Science and all procedures were performed in accordance with the Declaration of Helsinki of 1975, as revised in 2013. The nature of the present investigation was explained to the patients, and written informed consent was obtained.

The parameters assessed were (a) demographic variables - the demographic information collected from the patients were (1) age and (2) sex; (b) periodontal parameters - the periodontal examination was done by one trained investigator who was blinded to the study groups. The periodontal examination was performed using a Williams periodontal probe. Periodontal parameters included PI [10], GI [11], PPD, and CAL. The measurements were recorded to the nearest millimeter. The periodontal diagnosis of healthy and chronic periodontitis patients followed the guidelines presented by the 1999 International Workshop for a Classification of Periodontal Diseases and Conditions of the American Academy of Periodontology; (c) biochemical parameters - fasting blood sugar, glycated hemoglobin, serum creatinine, and eGFR levels were recorded for all the patients. To analyze the oxidative stress marker GPx, venous sample was collected and centrifuged at 5000 rpm at 4°C for 10 minutes using the Biofuge Stratos (Langenselbold, Germany: Thermo Electron LED GmbH), high-speed refrigerated microcentrifuge. After centrifugation, they were stored at -70°C until analysis. Oxidative stress biomarker serum glutathione peroxidase (GPx) concentrations were measured by a commercial enzyme-linked immunosorbent assay kit (catalog number: O13850). Reactions were read using a multimode micro/ELISA plate reader and plate washer (Varioskan Flash 2013; Thermo Fischer Scientific, Finland). The manufacturers’ instructions were followed for all kits used in this study.

The serum creatinine and fasting blood glucose levels were determined by an automated method, using semiautomated biochemical analyzer, CORALAB 3000 (Bambolim, India: Tulip Diagnostics). The eGFR was calculated based on serum creatinine values using CKD-EPI equation 2009. CKD was diagnosed with chronic kidney disease stages 2-4 (not on dialysis). Participants with GFR<90 mL/min/1.73 m^2^ constituted the control group. However, due to the small sample of the severely reduced GFR population, we combined mild, moderately, and severely reduced eGFR populations in one category, according to the renal function stages proposed by the National Kidney Foundation [12]. The C group consisted of periodontally healthy subjects seeking general master health checkups without signs and symptoms of renal disease having GFR>90 mL/min and any other systemic ailments. The CKD group consisted of patients with clinical diagnosis of renal failure, having GFR between 89 and 15 mL/min, CKD stages 2-4, and receiving conservative treatment (pre-dialysis). All subjects completed their personal, medical, and dental history questionnaires. Participants were informed of their randomization assignment at the baseline visit.

Statistical analysis

All statistical analysis was performed using Statistical Package for Social Science (SPSS) version 17 (Chicago, IL: IBM Corporation) for Microsoft Windows. The data were normally distributed. Descriptive statistics were presented as numbers and percentages. Mean and standard deviation for all the parameters (demographic, biochemical, and periodontal) were estimated for all the groups and the level of significance was determined. Chi-square test and one-way ANOVA were used to find the significant differences in GPx levels between the groups. Pearson correlation coefficient analysis was done to correlate the GPx with the other variables. A p≤0.05 was considered statistically significant.

## Results

The demographic variables were compared between the groups. On comparing the demographic variables, the mean age was found to be significantly higher in CKD+CP as compared to the others (61.47±10.99 years) (p<0.001). On gender comparison, males were found to be predominant in CKD and CKD+CP groups, whereas females were found to be more in C and CP groups. The gender proportions did not show any significant difference between the groups. The mean fasting blood sugar was high in group CKD+CP (130.03±52.07 mg/dL) when compared with other groups and was found to be statistically significant (p<0.001). The mean glycated hemoglobin was high in group CKD+CP (6.62±1.46%) when compared with other groups and was found to be statistically significant (p<0.001). Serum creatinine was found to be higher in the CKD group whereas eGFR was higher in the healthy individuals (C group) and this was found to be significant with p<0.001. The mean GPx value was higher in group CP when compared to other groups and was found to be statistically significant (p<0.001). The mean plaque index, gingival score, mean probing pocket depth (mm), mean proportion of sites with probing pocket depth more than 5 mm, mean clinical attachment loss (mm), mean proportion of sites with clinical attachment loss more than 3 mm was higher in the CKD+CP group when compared to the other groups (Table 1).

**Table 1:** Intergroup comparison of the demographic, biochemical parameters, and periodontal parameters assessed among C, CP, CKD, and CKD+CP groups. *Significant value. **Non-significant value. C: control; CP: periodontitis and non-CKD patients; CKD: chronic kidney disease; FBS: fasting blood sugar; HbA1c: hemoglobin A1C; eGFR: estimated glomerular filtration rate; GPx: serum glutathione peroxidase; PI: plaque index; GI: gingival index; PPD: probing pocket depth; CAL: clinical attachment loss

Variable	C (30)	CP (30)	CKD (30)	CKD+CP (30)	ANOVA	p-Value
Age (years) (mean±SD)	37.63±10.26	54.03±9.10	59.27±10.90	61.47±10.99	32.56	0.00^*^
Male (%)	22.2	20.6	27	30.2	0.39	0.173^**^
FBS (mg/dL)	89.43±9.72	111.17±48.08	103.73±41.57	130.03±52.07	5.008	0.003^*^
HbA1c (%)	5.28±0.22	5.97±1.16	5.77±1.22	6.62±1.46	7.439	0.00^*^
Serum creatinine (mg/dl)	0.80±0.14	0.72±0.11	1.20±0.43	1.08±0.19	25.20	0.00^*^
eGFR (mL/min)	107.67±14.81	101±7.10	64.77±18.94	68.43±12.45	74.154	0.00^*^
GPx	8.28±4.3	35.58±25.09	26.25±20.19	26.74±15.99	12.03	0.00^*^
PI	0.79±0.29	1.79±0.22	0.88±0.29	2.27±0.23	225.748	0.00^*^
GI	0.91±0.34	1.92±0.35	1.05±0.30	2.42±0.18	172.28	0.00^*^
PPD (mm)	1.33±0.17	2.77±0.27	1.29±0.09	3.18±0.24	675.859	0.00^*^
% sites with PPD>5 mm	0±0	12.03±9.29	0±0	21.87±7.49	94.276	0.00^*^
CAL (mm)	0±0	0.72±0.26	0±0	1.14±0.48	126.427	0.00^*^
% sites with CAL>3 mm	0±0	19.36±7.7	0±0	30.44±11.00	150.83	0.00^*^

The mean proportion of subjects with GPx ranges between 31-60% and 16-30% was higher in group CKD+CP. On comparing the mean proportion of subjects within GPx values 0-15%, it was higher in the C group. GPx values within 61-90% were found to be higher in the CP group (p<0.001) (Table 2). Overall Pearson's correlation analysis was done to assess the relationship between all the study variables and GPx. A significant correlation was found between GPx and PI, GI, PPD, and CAL (Table 3).

**Table 2:** Percentage proportion of subjects among C, CP, CKD, and CKD+CP groups with serum GPx level in various ranges. *Significant value. C: control; CP: periodontitis and non-CKD patients; CKD: chronic kidney disease; GPx: serum glutathione peroxidase

Variable	C (%)	CP (%)	CKD (%)	CKD+CP (%)	Chi-square	p-Value
GPx 0-15%	50.9	12.3	19.3	17.5	46.595	0.000^*^
GPx 16-30%	3.7	33.3	29.6	33.3		
GPx 31-60%	0	32.1	28.6	39.3		
GPx 61±90%	0	62.5	37.5	0		

**Table 3:** Overall Pearson correlation between glutathione peroxidase and various parameters. *Non-significant value. **Significant value. ***Highly significant value.

Variables	Glutathione peroxidase (GPx)	
	Pearson correlation	p-Value
Serum creatinine value (mg/dL)	0.001	0.988*
Fasting blood sugar value (mg/dL)	0.097	0.294*
Glycated hemoglobin score (%)	0.151	0.1*
Plaque index score	0.215	0.018**
Gingival index score	0.229	0.012**
Mean probing pocket depth (mm)	0.245	0.007***
Mean clinical attachment loss (mm)	0.211	0.021***

## Discussion

In periodontitis, the inflammatory process extends from the gingiva to the deeper connective tissues inducing alveolar bone loss and periodontal ligament destruction as a result of host-derived matrix metalloproteinase synthesis and activation. The ulcerated gingival epithelium migrates apically, lining the periodontal pocket around the affected tooth. Periodontal bacteria and their by-products can migrate into the systemic circulation through the blood vessels and other fluid channels to reach the distant sites, thereby contributing towards the systemic illness [1]. Periodontitis and CKD share common etiopathogenic mechanisms such as a common inflammatory status, presence of periodontal bacteria in the blood which may injure the nephrons, and diabetes and hypertension where bacteria, their endotoxins, and the ensuing cascade of immunoinflammatory reaction simulate each other [1,3].

Oxidative stress, which is an imbalance between free oxygen radicals, commonly referred to as the reactive oxygen species (ROS), and antioxidant defense system, is capable of causing damage to various cellular and extracellular components and has shown to play a major role in various inflammatory diseases [13]. The ROS produced by activated neutrophils and other defense cells in response to periodontal bacteria or bacterial metabolites such as LPS contributes to local periodontal tissue destruction [14]. The local and systemic increase in the reactive oxygen species or reactive nitrogen species (RNS) during periodontitis alters the oxidant to the antioxidant ratio in the body [15]. However, the role of oxidative stress in CKD patients with periodontitis as such still remains disputable [16].

Glutathione metabolism is one of the most important antioxidative defense mechanisms against oxidative stress in our body, as well as the periodontium [17]. The increase in antioxidant GPx levels in periodontitis occurs in response to the oxidative stress in the inflamed periodontal tissues [13]. Keeping the above facts in mind, the current study investigated the role of glutathione peroxidase as an antioxidant marker in periodontitis patients with and without CKD and compared them with the healthy controls to confirm that oxidative stress has a possible hidden link in stimulating periodontal disease and CKD.

The current study consisted of 120 patients categorized into four groups, including the C group (30 subjects), CP group (30 patients), CKD group (30 patients), and CKD+CP (30 patients). Demographic variables, biochemical and periodontal parameters were evaluated. GPx levels were also assessed among the groups. Among the demographic variables, age was significantly higher in the CKD+CP group, which was in accordance with studies by Baioni et al. and Mahendra et al. who stated that the prevalence and adverse events associated with both CP and CKD was found to be higher and positively associated with older individuals (Table 1) [18,19]. Thus, from the above findings, it can be postulated that the patients have prolonged exposure to their etiology as age increases, and thus the predisposition to periodontal as well as CKD also increases. It clearly denotes that age could be a significant confounder for periodontitis and CKD. On comparing the gender, male and female participants were more or less similar indicating that the diseases can be equally distributed among the sexes.

On comparing biochemical parameters, such as FBS, HbA1C, and serum creatinine, they were found to be higher in the CKD+CP group (Table 1). This was in accordance with results of Ricardo et al., Yoshihara et al., and Sharma et al. [20-22]. The authors attributed their results to the inflammatory nature of both CKD and periodontal disease, which may result in peripheral insulin resistance, leading to high glucose levels in the blood [20,21]. Key periodontal bacteria are capable of attaching to the nephrons and initiate an environment of inflammation by activating the host proinflammatory mediators which further result in damage of the nephrons, leading to renal insufficiency, culminating in the insufficient clearance of creatinine from the blood, resulting in increased serum levels of creatinine [22]. Thus, it is plausible to assume that the inflammatory response induced by periodontal disease could add to the total inflammatory burden in CKD patients and have an impact on the biochemical parameters [23].

Mean GPx values were higher in the CP group, followed by CKD and CKD+CP groups (Table 1). The results were in harmony with Wei et al. and Hendek et al. who demonstrated that the balance between oxidative stress and antioxidants is associated with occurrence, severity, and progression of periodontal disease [1,24]. The result of our study can be attributed to the tissue-protective and adaptive mechanisms. Our study is one of the very few studies to analyze the percentage proportion of subjects with various ranges of GPx and to correlate the levels of GPx with all the study variables. In the current study, the mean proportion of subjects with GPx ranges between 31-60% and 16-30% was higher in the CKD+CP group. On comparing the mean proportion of subjects within GPx values 0-15%, it was higher in the C group. GPx values within 61-90% were found to be higher in the CP group (p<0.001) (Table 2). Also, GPx was found to be positively correlated with plaque index, gingival index, probing depth, and clinical attachment loss (p<0.05) (Table 3). Hence, we infer that GPx is an antioxidant marker induced by these diseases. The antioxidant glutathione peroxidase exerts its action by inhibiting the NF-kB pathway, an attempt made to overcome the reactive oxygen species, thus alleviating the oxidative stress. Hendek et al. also indicated that increased levels of GPx in individuals with inflamed periodontal tissues could be a response to the oxidative stress present in the tissues due to active periodontal breakdown [24].

When the periodontal parameters such as PI, GI, PPD, CAL, percentage of sites with periodontal pockets, and CAL were compared between the groups, all of the periodontal parameters were significantly higher in the CKD+CP group. This was in accordance with results of Baioni et al. who suggested that renal tissue damage is linked to the severity of periodontitis, where large amounts of periodontal bacteria such as *Porphyromonas gingivalis, Tannerella forsythia, Fusobacterium nucleatum, Aggregatibacter actinomycetemcomitans, *and their toxins such as gingipains, leukotoxin A, outer membrane vesicles, and cytolethal distending toxin are capable of invading the renal tissues, resulting in oxidative imbalance in the kidneys [18].

It is stated that ROS are associated with oxidative stress and long-lasting systemic oxidative stress, which is thought to cause multi-organ failure [25]. If the increased levels of ROS overwhelm the antioxidant systems, the periodontium may be damaged which may influence the kidneys [1]. The defense cells release excessive amounts of ROS, contributing to the generation of lipid peroxide in the kidney [26]. Possible pathophysiological mechanisms in periodontal disease and high blood pressure also include oxidative stress [26,27]. As inflammation and oxidative stress play a role in the pathobiology of periodontitis and CKD, these diseases may amplify adverse outcomes when occurring concomitantly [28,29].

Overall, the current study gives a telescopic understanding of oxidative stress and its impact on periodontal and chronic kidney diseases. Our study confirms the role of glutathione peroxidase as an antioxidative biomarker in periodontitis patients with and without CKD which opens a new gateway for perio-systemic continuum. The present study is one of the very few studies in literature that assessed samples from pre-dialysis CKD patients with and without periodontitis, and also correlated the levels of the antioxidant enzyme, GPx with demographic, periodontal, and biochemical parameters. Also, another strength of the current study is the comprehensive assessment of periodontal disease, where all six sites around each tooth in the dentition of CKD patients were examined.

However, this is preliminary work with few limitations. First, this study was a cross-sectional study design with limited number of study participants. Second, periodontitis is a risk factor for the initiation of renal inflammation and so further longitudinal and randomized trials in humans are necessary to strengthen the role of oxidative stress as a risk for chronic kidney disease.

## Conclusions

Thus, from our study, it can be concluded that glutathione peroxidase levels were higher in the group containing periodontitis patients among the four groups, thus, proposing a new pathogenic mechanism linking the risk of chronic kidney disease and periodontitis by the influence of oxidative stress. This proposed link is reinforced by the positive correlation between GPx and the periodontal parameters. Personal oral health care, routine dental check-ups, and professional oral hygiene maintenance are efficient in controlling the oral infection and minimizing the oxidative stress and spreading of systemic inflammation from periodontitis. Healthcare providers must perform comprehensive patient management across disciplines and individual specialties.
